# Distinguishing the victim from the threat: SNP‐based methods reveal the extent of introgressive hybridization between wildcats and domestic cats in Scotland and inform future in situ and ex situ management options for species restoration

**DOI:** 10.1111/eva.12720

**Published:** 2018-12-19

**Authors:** Helen V. Senn, Muhammad Ghazali, Jennifer Kaden, David Barclay, Ben Harrower, Ruairidh D. Campbell, David W. Macdonald, Andrew C. Kitchener

**Affiliations:** ^1^ RZSS WildGenes Laboratory, Conservation Department Royal Zoological Society of Scotland Edinburgh UK; ^2^ Conservation Department Royal Zoological Society of Scotland Edinburgh UK; ^3^ Scottish Natural Heritage Inverness UK; ^4^ Wildlife Conservation Research Unit, Zoology, Recanati Kaplan Centre, Zoology Department Oxford University Oxford UK; ^5^ Department Natural Sciences National Museums Scotland Edinburgh UK; ^6^ Institute of Geography, School of Geosciences University of Edinburgh Edinburgh UK

**Keywords:** captive populations, carnivores, conservation management, invasive species

## Abstract

The degree of introgressive hybridization between the Scottish wildcat and domestic cat has long been suspected to be advanced. Here, we use a 35‐SNP‐marker test, designed to assess hybridization between wildcat and domestic cat populations in Scotland, to assess a database of 295 wild‐living and captive cat samples, and test the assumptions of the test using 3,097 SNP markers generated independently in a subset of the data using ddRAD. We discovered that despite increased genetic resolution provided by these methods, wild‐living cats in Scotland show a complete genetic continuum or hybrid swarm structure when judged against reference data. The historical population of wildcats, although hybridized, clearly groups at one end of this continuum, as does the captive population of wildcats. The interpretation of pelage scores against nuclear genetic data continues to be problematic. This is probably because of a breakdown in linkage equilibrium between wildcat pelage genes as the two populations have become increasingly mixed, meaning that pelage score or SNP score alone is poor diagnostic predictors of hybrid status. Until better tools become available, both should be used jointly, where possible, when making management decisions about individual cats. We recommend that the conservation community in Scotland must now define clearly what measures are to be used to diagnose a wildcat in the wild in Scotland, if future conservation action is to be effective.

## 1 INTRODUCTION

Hybridization between a native and a non‐native species creates a serious challenge to conservation management. This is because hybridization blurs the boundary between what we are trying to protect (native species) and the threat (non‐native species). If the extent of hybridization between the two species is advanced, and the management measures taken to eradicate hybrids are stringent, then the potential exists to unintentionally remove a large proportion of the genome of the threatened species. Stringent action will remove adaptive variation, which will be disadvantageous for the species' survival on an evolutionary timescale (Sgrò, Lowe, & Hoffmann, 2011; Spielman, Brook, & Frankham, 2004). In the extreme, management measures could drive population numbers down so severely, so as to threaten the population demographically. On the other hand, if the measures taken to eliminate hybrids are too weak/lenient, and animals with a large proportion of introgressed genome are allowed to breed, then costly conservation management actions are likely to have little impact on improving the *status quo* and all that will be conserved is a hybrid swarm. In either scenario, hybridization may impact on the population's fitness, so that it collapses in an extinction vortex (Caughley, 1994; Fagan & Holmes, 2006; Gilpin & Soulé, 1986). On the other hand, hybrids may have greater fitness than either parent species in anthropogenic landscapes making conservation of the native parent species more difficult (Lehman et al., 1991; Seehausen, Takimoto, Roy, & Jokela, 2008; Stelkens et al., 2014).

### 1.1 The genetic management of hybridization

Management of hybridization requires tools to distinguish hybrids from non‐hybrids. This is not straightforward, and our ability to do this is intrinsically linked to the methods chosen to measure hybridization. Molecular genetic tools have been commonly used to do this, primarily through the use of nuclear unlinked molecular genetic data (microsatellites and SNPs), followed by statistical assignment methods (most commonly STRUCTURE, (Pritchard, Stephens, & Donnelly, 2000; Falush, Stephens, & Pritchard, 2003; Falush, Stephens, & Pritchard, 2007); NewHybrids, (Anderson & Thompson, 2002); and BAPS (Corander, Waldmann, & Sillanpa, 2003)). However, a combination of other markers such as mtDNA, sex‐linked markers and phenotypic traits may be used in addition to such nuclear marker data. The advent of genomewide (linked marker) data and whole genome data opens up possibilities of in‐depth screening and reconstruction of ancestry and admixture (Hellenthal, Auton, & Falush, 2008; Lawson, Hellenthal, Myers, & Falush, 2012; Malinsky, Trucchi, Lawson, & Falush, 2016; Price et al., 2009). It also potentially opens up the possibility, through selective breeding, of the elimination of large tracts of introgression (Amador, Fernández, & Meuwissen, 2013; Amador, Hayes, & Daetwyler, 2014).

The basic principle of nuclear DNA hybrid testing is to survey the genome of an individual and estimate what proportion has been inherited from each parent species (its hybrid score, henceforth *Q,* (Pritchard et al., 2000)). At a conceptual level, this approach is relatively easy to understand, but in practice there are two issues which complicate matters and make hybridization a difficult genetic phenomenon, for which to assay.

1. By definition, hybridizing species are closely related (especially in the case of wild progenitor–domestic interactions (Randi, 2008)), and therefore much of their genome will be genetically indistinguishable from each other. Thus, if the approach is limited to a small number of markers, the first step is generally to try and find genetic markers that differentiate between the parent species and use this marker set to assay for hybridization. Therefore, the reliability of the marker set will be intrinsically linked to the quality of the reference data used to generate it. Since it is not always easy to find reliable reference individuals that do not have hybrid ancestry, this can be a complicating factor that introduces circularity and a level of uncertainty (Randi, 2008; Senn & Pemberton, 2009). The larger the number of sites in the genome (markers) that we can use to examine hybridization, the less reliant we are on any one marker and possible associated anomalies in the reference datasets, or effects of ancestral polymorphism. Approaches like those implemented within Bayesian assignment software (e.g., STRUCTURE and BAPS), which do not require the definition of reference individuals and which tolerate a degree of ancestral polymorphism due to the use of linkage disequilibrium as the primary model, also offer resilience to this (Bohling, Adams, & Waits, 2013; Putman & Carbone, 2014; Vähä & Primmer, 2006).
2. Assuming introgression progresses through the generations by backcrossing (as it will do in recently hybridizing populations (Goodman, Barton, Swanson, Abernethy, & Pemberton, 1999)), the proportion of the genome that has introgressed in any one individual reduces by, on average, ½ every generation (although there is considerable variation surrounding this (Boecklen & Howard, 1997)). This means that the more distant the hybrid ancestry of an individual is, the more difficult it is to detect. Very large numbers of genetic markers are required to detect distant hybrid ancestry reliably and estimate accurately the proportion of the genome that is introgressed. This means that it is much harder to understand situations where hybridization has happened between the parent species many generations ago or where a collapse into a hybrid swarm (Mayr, 1963) has occurred.

An implementable hybrid test is a balance between the ideal (many markers, ideally whole genome data (e.g., Amish et al., 2012; Nadeau et al., 2012; Kawakami et al., 2014; Ai et al., 2015; Li, Davis, Eizirik, & Murphy, 2016; Cahill et al., 2017; Nelson, Wallberg, Simões, Lawson, & Webster, 2017) and the necessary practical constraints of sample quality, cost and turnaround time required for running that test in a management situation.

### 1.2 Hybridization and the wildcat

Hybridization with domestic cat is an important threat to the wildcat, *Felis silvestris*. To date, many genetic studies have been conducted on wildcat hybridization worldwide using a variety of both molecular genetics and statistical assignment methods (e.g., Beaumont et al., 2001; Daniels et al., 2001; Driscoll, Yamaguchi, O'Brien, & Macdonald, 2011; Eckert, Suchentrunk, Markov, & Hartl, 2010; Hartmann, Steyer, Kraus, Segelbacher, & Nowak, 2013; Krüger, Hertwig, Jetschke, & Fischer, 2009; Le Roux, Foxcroft, Herbst, & MacFadyen, 2014; Mattucci et al., 2013; Nussberger, Currat, Quilodran, Ponta, & Keller, 2018; Nussberger, Greminger, Grossen, Keller, & Wandeler, 2013; Nussberger, Wandeler, Weber, & Keller, 2014; O'Brien et al., 2009; Oliveira, Godinho, Randi, & Alves, 2008; Oliveira et al., 2015; Pierpaoli et al., 2003; Steyer, Tiesmeyer, Muñoz‐Fuentes, & Nowak, 2018). In Britain, it is the only surviving native felid. Once widespread, it has been driven to near extinction by a combination of threats, which include habitat loss, persecution and hybridization with and disease transfer from the domestic cat (*Felis catus*; Macdonald, Daniels, Driscoll, Kitchener, & Yamaguchi, 2004; Macdonald et al., 2010; Macdonald & Loveridge, 2010). Now, its range is restricted to the Highlands of Scotland, north of the “Central Belt” running between Glasgow and Edinburgh (Macdonald et al., 2004; Davies and Gray, 2010). The two species are estimated to have been separated from each other for at least 1.1my (Li et al., 2016), revising previous estimates of>250,000 years (Driscoll et al., 2007). Domestic cats have been present in Britain for over>2000 years (O'Connor & Kitchener, 2010), and so opportunity for hybridization with wildcat has existed since then, although it has been argued that its contribution would have been low until relatively recently (Kitchener, 1998), but see also Daniels, Balharry, Hirst, Kitchener, and Aspinall (1998).

The wildcat in Scotland, or Scottish wildcat*,* is a subpopulation of the European wildcat based on the current taxonomic consensus (Kitchener, Breitenmoser, Eizirik, & Werdelin, 2017), although it has been described as a subspecies *Felis silvestris grampia* Miller, 1912. Estimates of genetic divergence between wildcat in Scotland and continental Europe do not currently exist (see Neaves & Hollingsworth, 2013 for an initial exploration of haplotype differences). Although the European wildcat is classified as least concern (LC) globally according to the International Union for the Conservation of Nature [IUCN] Red List (assessment published in 2015), it is listed on Annex IV of the EU Birds and Habitats Directive, the assessment of the conservation status for the UK is “bad,” and the trend is “declining” European Community Directive on the Conservation of Natural Habitats and of Wild Fauna and Flora (92/43/EEC).

In 2004, extrapolation from a variety of data available at the time led to a suggestion that of an estimate of 3,500 wild‐living cats across Scotland, only 400 individuals would be likely to be considered phenotypically wildcat based on “classic pelage characteristics” (Macdonald *et al*., 2004). A decade later, extrapolations from camera‐trapping data carried out across Northern Scotland over 23 sites by Kilshaw (2015), encompassing 32,732 trapping days resulted in an estimate of 115–314 cats which display wild or mostly wild pelage traits (a score of 14/21 or more on the 7PS pelage scoring system of (Kitchener, Yamaguchi, Ward, & Macdonald, 2005)). These numbers are framed against many wild‐living feral cats or hybrids and were estimated from a subset of 15 of the 23 sites surveyed. Of these sites, 16% of cats caught on camera were deemed to be wildcats, 23% were hybrids, and 60% were feral/domestic Kilshaw (2015). The seminal genetic study of wildcats in Scotland by Beaaumont et al. (2001), based on analysis of 230 wild‐living cat samples (collected mostly between 1989 and 1994) at nine microsatellite loci, estimated that 41% of cats sampled in the wild could be wildcats. A further 42% were deemed to be hybrid and 17% domestic (Beaumont et al., 2001). In surveys covering nine sites to scope out the priority areas for the Scottish Conservation Action Plan (SNH, 2014, of 45 individuals seen on 7,493 trap nights, only six could be wildcat based on strict phenotype (a score of 19/21 or more on the 7PS), 24 were hybrid, and 15 were domestic. Lowering the definition to the relaxed pelage criteria with a 7PS score of ≥14 also used by Kilshaw (2015) and Kilshaw, Johnson, Kitchener, and Macdonald (2015) changed the estimate to 22 wildcat individuals and eight hybrids. A clear issue with estimating wildcat numbers, aside from the challenges and costs involved with monitoring an elusive felid in the field, is the lack of consensus in standardizing a definition and difficulty of aligning the results of different survey methods (Neaves & Hollingsworth, 2013; Yamaguchi, Kitchener, Driscoll, Ward, & Macdonald, 2004). This lack of standardization of methods and definitions makes comparisons between studies to demonstrate population and introgression trends impossible.

Conservation action for the wildcat in Scotland is now being coordinated according to a National Action Plan (https://www.scottishwildcataction.org/). This plan, which has been agreed by 23 partner organizations, is running from 2015 to 2020 and includes Trap‐Neuter‐Vaccinate‐Return (TNVR) measures for feral cats and individuals that exhibit obvious signs of hybridization in the six priority areas selected on the basis of a high probability of high wildcat population density (SNH, 2014, Commissioned Report No. 768), an education programme surrounding responsible cat ownership, aimed at reducing the number of un‐neutered domestic house cats in wildcat areas, various field research projects to increase knowledge of the species in the field and a strengthening of ex situ conservation measures for wildcats, both by assessing animals currently in captivity and scoping additional founders from outside priority areas and through semen banking. Captive breeding for reintroduction and reinforcement is a well‐established conservation measure (IUCN/SSC, 2013; McGowan, Traylor‐Holzer, & Leus, 2017) and release of captive‐bred animals is a potential conservation intervention that needs to be considered, providing there are suitable areas free of threat (particularly the threat from feral cats and obvious hybrids). Since the publication of the “IUCN Policy Statement on Captive Breeding” (1987) by the IUCN‐SSC Captive Breeding Specialist Group (CBSG), it has been recommended that when the population of vertebrate taxa falls below one thousand individuals, swift cooperation between field conservationists and captive breeding specialist's takes place.

Two primary means of distinguishing wildcats are currently used in the implementation of the Conservation Action Plan, the 7PS pelage scoring test of Kitchener et al. (2005), where 17/21 is taken as the cut‐off for a wildcat individual, and a *genetic* test based on 35 nuclear SNP markers (Senn & Ogden, 2015). However, note that Kitchener et al. (2005) used a score of 19 and above to define a wildcat, so that some degree of introgression has been accepted for pragmatic management reasons, with only a limited impact on external morphology.

The pelage and SNP test are used in tandem for ex situ assessments (Senn & Ogden, 2015), whereas the pelage test alone is currently used for practical reasons when implementing in situ conservation measures (TNVR), although TNVR actions are being monitored retrospectively via the SNP test.

Here, we present the results of the screening of a sample of contemporary and historical wild‐living animals and the entire ex situ population. This enables us to understand the current and historical situation for wildcat hybridization in Scotland as a basis for management decisions going forward. We also explore the robustness of the common approaches used to understand hybridization. We do this by comparison of hybrid scores obtained on the 35 SNP system of Senn and Ogden (2015) to data at >3,000 SNPs generated independently via ddRAD analysis. We also do this via exploration of the relationship of the 7PS pelage scoring test of Kitchener et al. (2005) to the 35 SNP genetic system.

## 2 METHODS

### 2.1 Production of datasets used in the paper

#### 2.1.1 35 SNP dataset

This dataset consists of 295 individuals, which have been typed at 35 SNP loci drawn from Nussberger et al. (2013) that have been identified as discriminatory between the following two groups; [(Scottish+mainland European wildcats) vs. (UK+mainland European domestic cats)]. Justification for the 35 SNP panel used is laid out in detail in Senn and Ogden (2015). Wet laboratory methods consist of PCR amplification of the samples with 35 TaqMan SNP Probes on a StepOne platform (Senn & Ogden, 2015). This dataset was divided into a variety of sub‐datasets based on the different methodologies used to collect the samples. Since the focus of collection of wild‐living cats has shifted over time, this has been done to assist with understanding how collection bias might influence conclusions drawn from the results (more later). The datasets (summarized in Table 1) are as follows:

**Table 1:** Summary of the analysis results for each dataset. Cats with an LBQ_35 ≥ 0.75 are deemed to be wildcats for management purposes

Dataset	*N*	*N* (with pelage)	Dates of sample collection (mean of known dates)	Sample locality summary	Wildcat (LBQ ≥ 0.75)	Hybrid	Domestic (UBQ ≤ 0.25)	Mean 7PS pelage score (range)	Collection bias
35SNP_historical_cats	60	51	1895–1987 (1937)	Scotland wide (*N* of Highland Boundary fault)	54	5	1	18 (10.5–21)	Shot cats given to and purchased by museums—possibly biased towards cats that look like wildcats
35SNP_wildliving_dead_cats	125	33	1989–2016 (2007)	Scotland wide roadside (mostly *N* of Highland Boundary Fault)	21	87	17	12 (8–17)	Predominantly roadkill—bias difficult to determine (see discussion)
35SNP_wildliving_survey_cats	19	18	2013–2014 (2014)	SNH Priority Area Survey targeting wildcats	0	19	0	13 (7–20)	Survey of supposed wildcat areas—biased towards higher pelage score
35SNP_captive_cats	72	28	2017	Living captive wildcats	63	9	0	17 (11–20.5)	Captive cats—founders biased towards higher pelage score cats
35SNP_domestic_cats	19	n/a	2014	Edinburgh City Region	0	0	19	n/a	n/a
3097SNP_dataset	76	n/a	Subset of 68 cats from above data and eight additional cats collected across the UK	26	39	11	n/a	n/a	

##### 35SNP_HISTORICAL_CATS

A dataset of 60 cats collected between 1895 and 1985 by National Museums Scotland, Natural History Museum (London) and the New Walk Museum Leicester. These cats are primarily cats identified as wildcats that were shot by gamekeepers. The samples taken from these animals consisted of fragments of dried or tanned skins taken with sterile scalpels, which were then extracted with Qiagen Investigator Kits (Qiagen) according to manufacturer's instructions. Of these cats, 51 were scored for pelage characters, from the preserved skins by ACK.

##### 35SNP_WILDLIVING_DEAD_CATS

A dataset of 125 cats collected from ~1990–2015 by National Museums Scotland with the assistance of a wide variety of partners. The cats are primarily victims of road traffic accidents, although in some cases they were shot, or the fate of specimen is unclear. Note that it was not possible to include the samples from the Beaumont et al. (2001) study. Sample type consists of skeletal muscle tissue, which was extracted with Fuji film or Qiagen blood and tissue kits according to manufacturers' instructions. Of these samples, 33 have pelage scores taken from photographs of dead cats or their tanned skins by ACK.

##### 35SNP_WILDLIVING_SURVEY_CATS

This dataset contains 19 cats trapped as part of the Survey of the Priority Areas conducted prior to the initiation of the current action plan (SNH, 2014, Commissioned Report No. 768.). Sample type of these cats consists of EDTA blood extracted with Fuji film or Qiagen blood and tissue kits (Qiagen), according to manufacturers' instructions. Of these, 18 samples have pelage scores (following Kitchener et al., 2005), scored from photographs taken when the cat was under anaesthesia in the field under Scottish Natural Heritage animal licence number 21,463 and Animals Scientific Procedures Act personal licence number 70/25,690.

##### 35SNP_CAPTIVE_CATS

A dataset of 72 wildcats, which represented 100% of the potential breeding population within the UK captive breeding programme as of May 2017. Sample type for these cats consists of EDTA blood taken during routine health screening, extracted with Fuji film or Qiagen blood and tissue kits (Qiagen), according to manufacturers' instructions. Of these samples, 28 have pelage scores, taken from photographs taken when the cats were under anaesthesia for routine health screening (https://www.scottishwildcataction.org/media/42346/protocol-for-photographing-dead-or-anaesthetised-wildliving-cat-dr-andrew-kitchener.pdf).

##### 35SNP_DOMESTIC_CAT

A dataset of 19 domestic cat samples collected from across the City of Edinburgh region. Pelage data are not available for this dataset.

Two additional 35 SNP datasets are referred to. (a) The **35SNP_global_dataset**, which included all the above data and all the additional reference data used to design the test (Senn & Ogden, 2015). (b) the **35SNP_non‐ref_dataset**, which consists only of** 35SNP_wildliving_dead_cats**,** 35SNP_wildliving_survey_cats** and **35SNP_historical_cats**.

A full sample list can be found in the Supplementary Material 1.

##### DDRAD DATA (3,097 SNPS)

For a subset of 68 of the cats typed at 35SNPs and an additional eight cats collected from across the UK, ddRAD analysis of the samples was conducted using a modification of the protocol by Peterson, Weber, Kay, Fisher, and Hoekstra (2012), which is described in Bourgeois et al. (2018). A list of all used samples can be found in Supplementary Material 2. In short, DNA quality was assessed via agarose gel electrophoresis on a 1% gel and only non‐degraded DNA (as judged by a tight high molecular weight band against a lambda standard) was selected for the library preparation stage. It should be noted here that DNA quality requirements for this protocol preclude running the analysis on poor quality or degraded samples (e.g., most historical or non‐invasive sample types). DNA was quantified using a Qubit Broad Range dsDNA Assay (Thermo Fisher Scientific) and normalized to 7 ng/µl. Each sample was processed in triplicate or quadruplet to enhance evenness of coverage of samples within the library. Individual genomic DNA was restriction‐digested using both SbfI and SphI enzymes, and Illumina‐specific sequencing adaptors (P1 and P2) were then ligated to fragment ends. The pooled samples were size selected (320–590 bp fragments) by gel electrophoresis and PCR amplified (15 cycles), and the resultant amplicons (ddRAD library) were purified and quantified. Combinatorial inline barcodes (5 or 7 bases long), included in the P1 &P2 adaptors, allowed each sample replicate to be identified postsequencing. The ddRAD library was sequenced on the Illumina MiSeq Platform (a single paired‐end run; v2 chemistry, 2 × 160 bases). Positive control samples were run on all libraries.

The sequence data were quality assessed using FastQC (Andrew, 2010) and the reads demultiplexed by barcode and quality filtered using the process_radtags module (default parameters) of the stacks bioinformatics pipeline (Catchen Hohenlohe, Bassham, Amores, & Cresko, 2013). The retained reads were then trimmed to a standard 135 bases in length. Demultiplexed read files were concatenated into read files for each individual (three or four barcode combinations per individual, see above). Read 1 and Read 2 files were then joined into a single file per individual.

The individual data files were then processed using the denovo_map.pl module of Stacks (‐M 2, ‐n 1) to assemble and create a catalogue of genetic loci contained in the data. The Stacks scripts export_sql.pl (snps_l = 1, ‐F snps_u = 1, ‐F pare_l = 10, ‐F alle_u = 2) was then used to create a whitelist of all loci, which contained exactly one SNP with two alleles, where the minor variant was present in at least 10 samples. This whitelist was then used to filter the data with the populations function (‐r 0.75, ‐m 10) to create a final dataset, where at each locus a minimum of 75% of individuals had been typed with a depth of at least 10 reads. Further filtering was then conducted in PLINK to remove any loci at which there was >33% missing data. This generated a final data matrix, which consisted of a list of 3,097 variable SNPs typed in 76 cats: 20 captive, five domestic and 51 wild‐living. The overall % missingness in the data matrix was 6.4%. This dataset is referred to as the **3097SNP_dataset**. We consider this dataset to represent the most unbiased genetic data for the Scottish wildcat so far. The 35 SNP test was derived from European wildcat data, and thus although effort was made to minimize any impact of bias in subsequent re‐design of the test for Scotland (Senn & Ogden, 2015), there is the potential that hidden issues with reference data or sub‐structuring within the wildcat or domestic cat populations could have biasing consequences on this relatively small panel of markers. The purpose of this dataset here is primarily to verify the performance of 35SNP system.

A full sample list of all samples can be found in the Supporting Information (Table S1), and a summary of the datasets can be found in Table 1.

### 2.2 Statistical analysis

#### 2.2.1 Inference of hybrid scores

Hybrid scores (Q) were assigned to individual cats in both the 35SNP dataset of all wildcats (**35SNP_global_dataset**) and the ddRAD dataset (**3097SNP_dataset**) using structure 2.3.4 (Falush et al., 2003; Pritchard, 2010; Stephens, Smith, & Donnelly, 2001). For each of the two datasets, the analysis was run separately. The STRUCTURE model makes use of the observation that populations show two properties in their genetic data: (a) Hardy–Weinberg equilibrium and (b) linkage equilibrium. The Bayesian MCMC (Markov chain Monte Carlo) approach seeks to optimize individuals' genotypes into the best K genetic clusters that conform to these properties. The following (standard) model was used: 500,000 burn‐in, 1,000,000 MCMC reps, admixture model (infer alpha) and correlated allele frequencies model (Lambda = 1). Three replicates per analysis were conducted to verify the stability of the results, which were examined through plotting of resulting Q scores in Excel. The structure output from one of the three replicates is always presented, as the variability between replicates was very low. Results were estimated at *K* = 1–5. In each case, the 90% posterior credibility interval (CI) for each animal was estimated using the option “print credible regions,” giving three components to the hybrid score: Q = the hybrid score estimate ranging from 0 (domestic cat) to 1 (wildcat); LBQ = lower boundary of the 90% CI of the hybrid score Q; and UBQ = upper boundary of the 90% CI hybrid score. To avoid confusion, scores generated from the 35 SNP dataset are referred to as Q_35, LBQ_35 and UBQ_35, whilst scores generated from the 3097SNP_dataset are referred to as Q_3097, LBQ_3097 and UBQ_3097. Cats with a LBQ_35 ≥0.75 are currently deemed to be wildcats from an ex situ management perspective under the Scottish Wildcat Action Plan. This cut‐off was chosen based on the available distribution of hybrid scores at the time and a desire to balance the opposing threats of hybridization and inbreeding to the Scottish wildcat (Senn & Ogden, 2015). To generate Q_35, LBQ_35 and UBQ_35 scores for the datasets **35SNP_captive_cats, 35SNP_domestic_cat, 35SNP_historical_cats, 35SNP_wildliving_dead_cats** and **35SNP_wildliving_survey_cats,** the results were simply extracted from the STRUCTURE results from the **35SNP_global_dataset**. To generate results for the **35SNP_non‐ref_dataset**, the STRUCTURE analysis was repeated on this dataset alone, in the absence of any other data, using identical analysis parameters to those given above.

#### 2.2.2 Analysis of hybrid scores

Regression of hybrid scores against each other was conducted in R (version 3.4.0). To test differences in the distribution of Q_35 values, the Kolmogorov–Smirnov testing was conducted using the *ks.test* function of R. Fisher's exact testing of the occurrences of wildcats for wildcats (Q_35 ≥ 0.75) versus non‐wildcats (Q_35 < 0.75) was also conducted for some pairwise comparisons of datasets using the *fisher.test* function in R.

Linear models (GLM) were fitted to the data using the *glm* function of R. The dependent variable was Q_35, which was fitted as a logit‐transformed value following Beaumont et al. (2001). The explanatory variables fitted were as follows: (a) 7PS pelage score (**7PS**), a continuous measure which was were centred on the mean value (14.947) prior to inclusion in the analysis, so that in the presence of interactions, the coefficients for linear variables were evaluated at the mean level of the interacting term. Both the linear and quadratic terms were fitted; (b) **dataset**, a factor with four levels: **35SNP_domestic_cat**;** 35SNP_captive_cats**;** 35SNP_historical_cats**; and **35SNP_wildliving_dead_cats**. The interaction between **7PS** and **dataset** was also fitted, once it was established that that the terms were significant in the absence of the interaction.

The significance of terms in the model was evaluated through t‐statistics for each term. The significance of change in log‐likelihood (deviance) between the new and old models was evaluated against the chi‐squared distribution, at the exclusion of each term.

#### 2.2.3 Mapping

Cats with known locality (Supplementary Material 1) either had an associated grid reference, which was converted to a Northing and Easting, or only had a locality descriptor, in which case an approximate location was estimated with the aid of Google Maps. These were plotted using ARC GIS, overlapping points were manually jittered to facilitate ease of viewing, and no cats were moved across the boundaries of Wildcat Priority Areas.

#### 2.2.4 Pelage scoring

7PS scores were estimated for cats where good quality photographs were available, or where cat skins had been preserved, following the method of Kitchener et al. (2005), with a minor modification. Instead of scoring presence/absence of broken stripes, and presence of spots, on flanks and hindquarters together, the presence/absence of broken stripes and spots was scored separately for flanks and hindquarters. In practice, these characters (broken stripes and spots) are correlated with each other, so that scores did not vary between these ways of scoring these factors and it was practically easier to score each sector of the pelage separately, especially as the score for each sector (flanks and hindquarters) may be different.

## 3 RESULTS

### 3.1 Robustness of the 35SNP test and examination of the analysis assumptions

**Figure 1:**
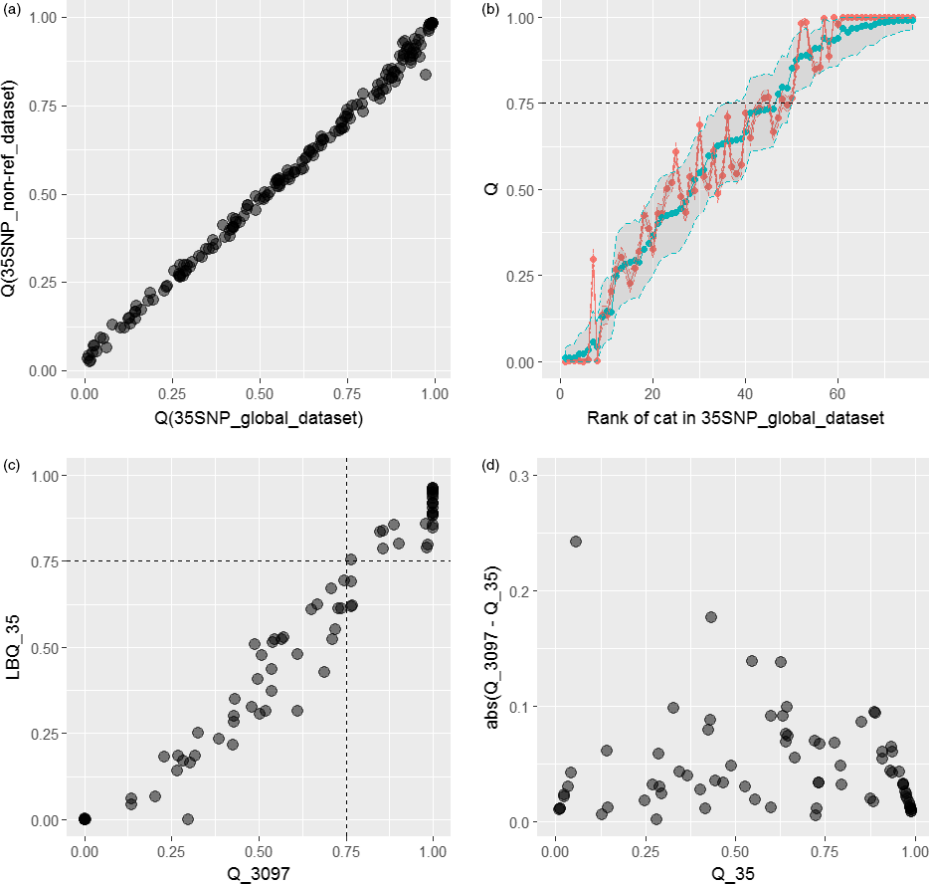
Verification of the 35SNP test. (a) Structure algorithm run only on the complete set of data of wild‐living cats (**35SNP_non‐ref_dataset**), in the absence of any other reference data captive or domestic, reveals that results still correlate highly with scores generated for the full dataset; thus, any potential effect introduced by inbreeding within the captive population or biases in the reference data can be ruled out. (b) Independent assessment of 76 cats using 3,097 SNPs gleaned from the ddRAD pipeline reveals that the majority of estimates fall within the 90% CI obtained from the original test. (c) And the management decision taken on the basis of the 35SNP test would not appear to differ greatly where the estimate from the larger 3097SNP dataset taken to be the true hybrid score (see in text for details).(d) A graphical presentation of the absolute difference in values obtained by the 3097SNP and 35SNP test against the values obtained by the 35SNP test illustrates in more detail the expected variance in the results

To check for the stability of the wild‐living results and to rule out biases that could be introduced by the choice of reference data and possible effects of inbreeding/drift within the captive population (Anderson & Dunham, 2008; Bohling et al., 2013; Vähä & Primmer, 2006); Rodriguez‐Ramilo & Wang 2012), the STRUCTURE algorithm was run only on the **35SNP_non‐ref_dataset**, in the absence of any other reference data, captive or domestic. The most appropriate value of K was 2 (Data S3). Regression of Q scores from the **35SNP_non‐ref_dataset** on Q scores from the **35SNP_global_dataset** revealed they were highly correlated, (Intercept = 0.018 ± 0.003, slope = 0.94 ± 0.005, *R* ^2^ = 0.995, *p* < 0.0001; Figure 1a).

Analysis of the **3097SNP_dataset** derived from the ddRAD analysis revealed that 69/76 (90.8%) of the estimates for Q fell within the original 90% CI of the estimate for Q derived from the 35 SNP panel (Figure 1b). Of the 27 cats classified as wildcats by the 35 SNP test (35SNP_LBQ≥0.75), all 27 (100%) were classified as wildcats using hybrid scores derived from the 3,097 SNP dataset (3097SNP_Q≥0.75). Of the 38 cats classified as hybrids by the 35 SNP test (35SNP_UBQ>0.25 and 35SNP_LBQ<0.75), three (7.9%) were classified as wildcats using hybrid scores derived from the 3,097 SNP dataset (3097SNP_Q≥0.75). These three cats were borderline with 3097SNP_Q of 0.764,0.766 and 0.767, respectively. Regression of Q_35 on Q_3097 revealed they were highly correlated (Intercept = 0.0096 ± 0.015, slope = 0.97 ± 0.021, *R* ^2^ = 0.964, *p* < 0.0001; Figure 1c).

### 3.2 Distribution of hybrid scores in the different sample populations of cats

**Figure 2:**
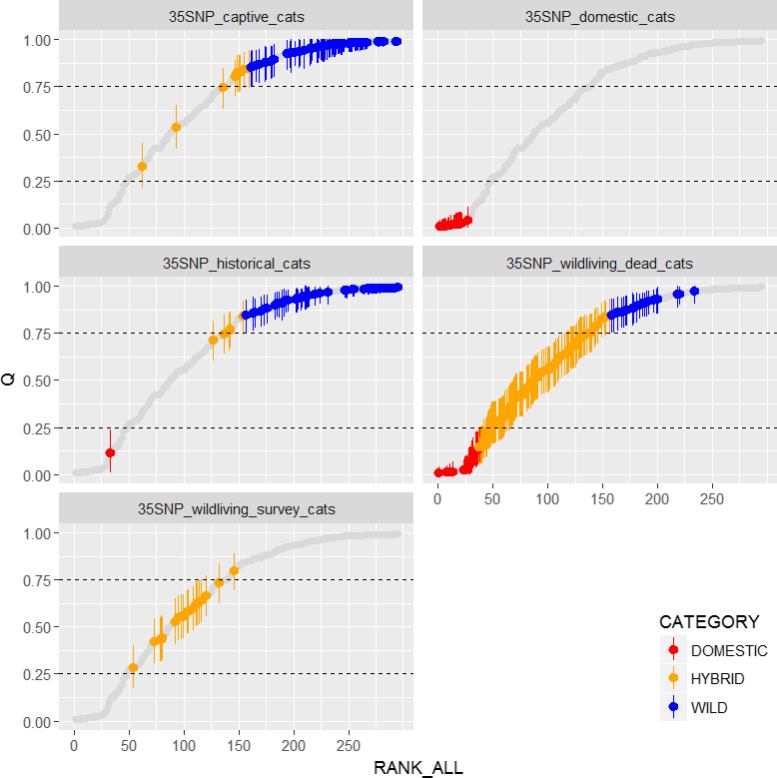
Hybrid scores obtained from the different datasets. Each score represents an individual cat. Each cat is given an estimated hybrid score Q by the software STRUCTURE with the limits of the lower and upper boundary of the 90% credibility interval marked with the vertical error bars. The scores have been ranked according to their position in the global dataset of all 35 SNP‐typed cats (all rankings are given as the grey shadow) and are presented in separate windows for each dataset. Cat that meet the 75% cut‐off for wildcat (LBQ ≥ 0.75) are presented in blue, those classed as hybrid are orange, and those with UBQ ≤ 0.25 are classed as domestic and are presented in red. Details of each dataset can be found in text

Structure analysis of the datasets **35SNP_wildliving_dead_cats** and **35SNP_wildliving_survey_cats** revealed a continuum of hybrid scores for cats sampled in the wild when judged against cats in captivity, **35SNP_captive_cats** and the historical wild dataset **35SNP_historical_cats** (Figure 2). The results are summarized by hybrid score in Table 1.

A variety of tests was conducted to investigate statistically how different the distribution of hybrid scores in the different datasets was: To understand whether the survey cats scores could be considered to be drawn from the same distribution as the roadkill cats scores, the following tests were run:

A two‐tailed Kolmogorov–Smirnov test revealed that the distribution of Q_35 in **35SNP_wildliving_survey_cats** was marginally different to that of **35SNP_wildliving_dead_cats** (*D* = 0.34737, *p*‐value = 0.03736). However, Fisher's exact testing of **35SNP_wildliving_dead_cats** against **35SNP_wildliving_survey_cats** for wildcats (Q_35 ≥ 0.75) versus non‐wildcats (Q_35 < 0.75) was not significant (odds ratio = 0, *p* = 0.0759).

To understand whether the distribution of historical cats scores differed significantly to that found in the wild in contemporary samples, the following tests were run: A two‐tailed Kolmogorov–Smirnov test revealed that the distribution of Q_35 in the **35SNP_historical_cats** dataset was significantly different to that of **35SNP_wildliving_dead_cats** (*D* = 0.740, *p*‐value < 0.001). Fisher's exact testing of **35SNP_wildliving_dead_cats** against **35SNP_historical_cats** for wildcats (Q_35 ≥ 0.75) versus non‐wildcat (Q_35 < 0.75) was highly significant (odds ratio = 0.023, *p* < 0.0001).

To understand whether the distribution of scores for captive cats differed significantly to that found in contemporary and historical samples, the following tests were run:

The distribution of Q_35 scores in the **35SNP_captive_cats** dataset was also significantly different to that of **35SNP_wildliving_dead_cats** (*D* = 0.75833, *p*‐value <0.001). The distribution of Q_35 scores in **35SNP_historical_cats** and **35SNP_captive_cats** was somewhat different from each other (*D* = 0.256, *p*‐value = 0.0279).

**Figure 3:**
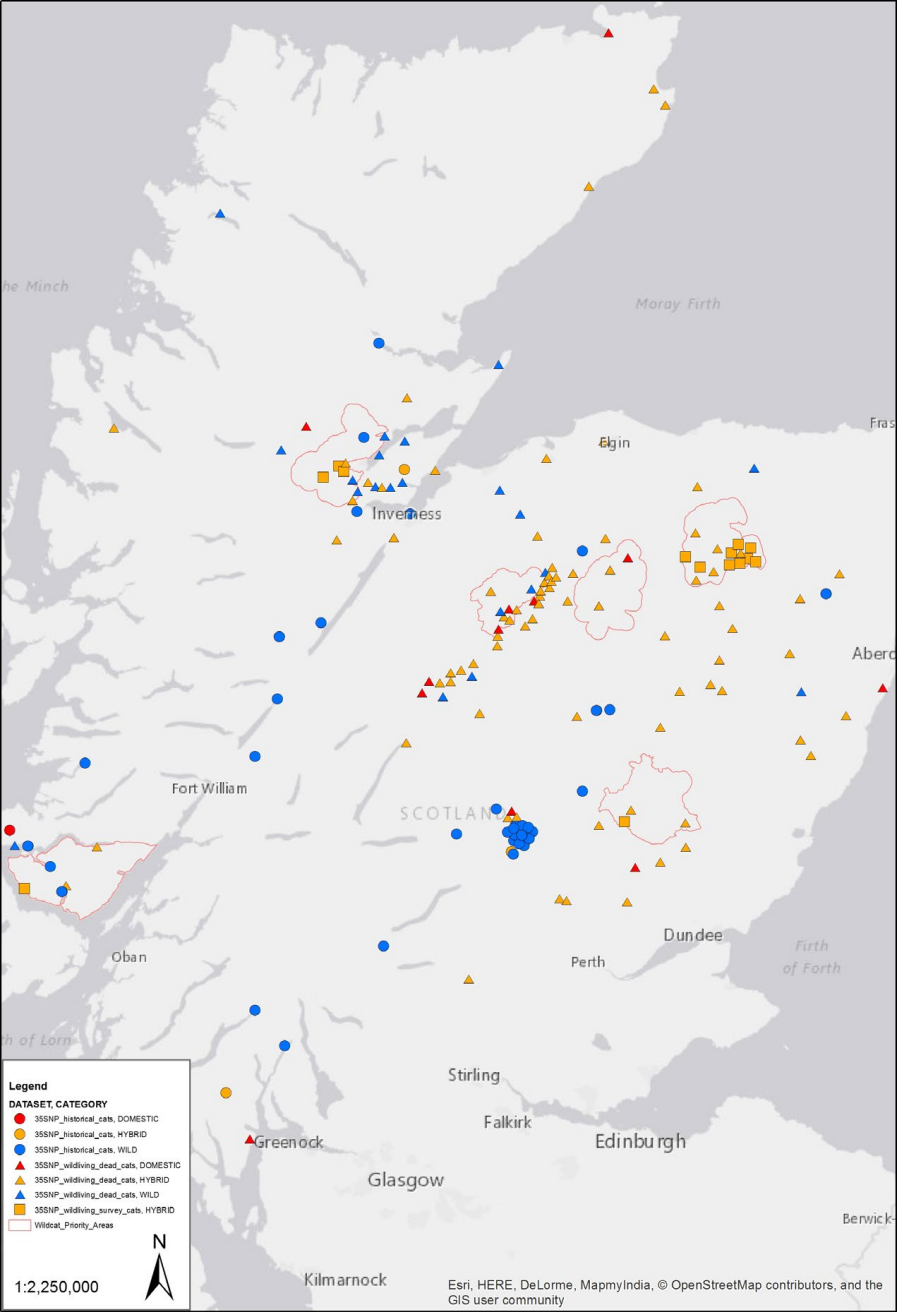
A map of Scotland with the locations of cats samples within the datasets **35SNP_historical_cats (circles)**,** 35SNP_wildliving_dead_cats (triangles)** and **35SNP_wildliving_survey_cats (squares)**. Where only a verbal location was given, the approximate location was chosen with the aid of Google Maps (e.g., the blue cluster in the map centre). Overlapping points were separated manually for ease of viewing, whilst respecting the boundaries of the Wildcat Priority Areas which are given in red. Points are coloured using the same genetic categories used for Figure 2

The 167 cats with geographical localities were mapped along with their hybrid scores (Figure 3).

### 3.3 Pelage and hybrid score

**Figure 4:**
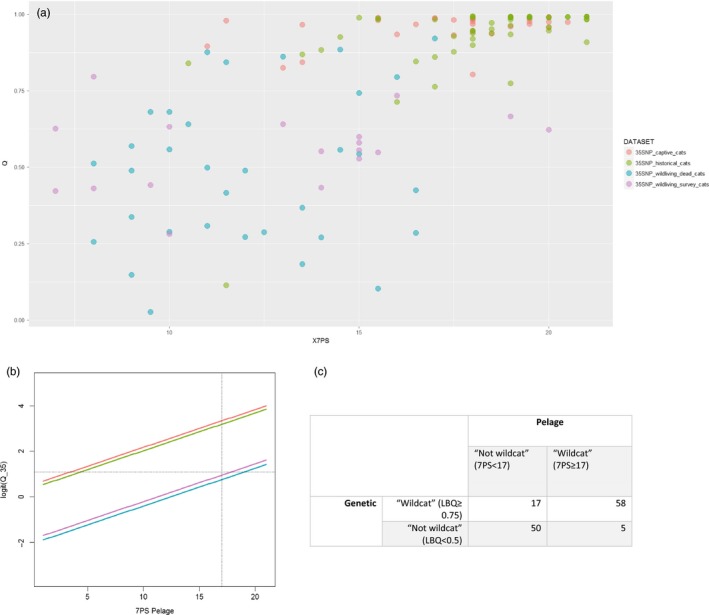
(a) The relationship between Q_35 and the 7PS pelage score. (b) Prediction from the model in Table 2 for logit‐transformed Q_35. Dashed lines represent the cut‐off values of Q = Logit(0.75) and 7Ps = 17 for genetic and pelage systems, respectively. (c) The number of individuals in the genetic and pelage categories for management purposes

The association between pelage and hybrid score in a dataset of 130 individuals can be found in Figure 4.

The final model of logit (Q_35) contained 7PS as a linear term and the factor **dataset**. Fitting the interaction between 7PS and dataset generated a marginal improvement in the model (Chisq = 10.905, *p* = 0.02352, *df *= 3), with the interaction 7PS*35SNP_historical_cats being marginally significant (est = 0.228 ± 0.11, *t* = 2.00, *p* = 0.048). The final model excluding the interaction is presented in Table 2.

**Table 2:** The model of logit (Q_35) with 7PS fitted as a linear term and dataset fitted as a factor. The explanatory variable 7PS was centred on it mean, and the factor levels are evaluated against the level DATASET35SNP_captive_cats

	Estimate	*SE*	*t*	*p*
Intercept	3.1081	0.2192	14.180	
7PS (mean centred)	0.1657	0.0362	4.576	<0.0001
DATASET:35SNP_historical_cats	−0.1500	0.2670	−0.562	0.575
DATASET:35SNP_wildliving_dead_cats	−2.5804	0.3419	−7.547	<0.0001
DATASET:35SNP_wildliving_survey_cats	−2.3851	0.3736	−6.385	<0.0001

## 4 DISCUSSION

### 4.1 The status of wildcat hybridization in Scotland

The study we present here confirms that hybridization between wild‐living wildcats and domestic cats in Scotland is extensive (Figure 2). Using a 35‐locus SNP test, whose resultant hybrid scores correlate highly with those generated independently from over >3,000 unbiased loci obtained via ddRAD analysis, we demonstrate that contemporary wild‐living cat populations within Scotland consist of a genetic continuum between *Felis silvestris and Felis catus* and that this was not the historical situation. The study of hybridization is constantly mired in issues surrounding certainty of baseline and thus potentially circularity (discussed in the introduction of Estoup, Cornuet, Rousset, & Guyomard, 1999). Hybridization between wildcats and domestic cats in Scotland appeared to be pervasive in previous studies using nine microsatellite loci (Beaumont et al., 2001) and 14 SNP markers (SNH, 2014, Commissioned Report No. 768.) and has long been apparent in phenotypic measures (Daniels et al., 1998; Yamaguchi et al., 2004), but discussion around historical baselines and the consequent power of the methods used to determine hybridization has remained (e.g., Neaves & Hollingsworth, 2013) and thereby dogged effective implementation of management action.

This study demonstrates that contemporary populations of wild‐living cats can be considered to be a hybrid swarm (Mayr, 1963) of genetically intermediate types and do not appear to display the more bimodal distribution of hybrid scores attributably to systems where hybridization is rare (e.g., as displayed in *Cervus elaphus* and *C. nippon* in Scotland with some localized exceptions (Goodman et al., 1999; Senn & Pemberton, 2009; Smith et al., 2018). A question to be resolved in more detail is what the spatial and temporal patterns of the progression of hybridization have been.

Clearly, it is very hard to unpick the effects of sampling bias, since the motivations for the collection of samples have varied over the years. The **35SNP_historical_cats** are cats that were shot or trapped in deliberate attempts to target wildcats by gamekeepers, whereas **35SNP_wildliving_dead_cats** mostly represent road traffic accident victims. Perhaps, on crossing any given stretch of road wildcats and domestic cats are equally likely become a fatality, but cats with wildcat‐like tabby pelage may be more likely to be collected by the volunteers that have handed the samples in (biasing the sample towards wildcats and hybrids). Conversely more hybridized and domestic cats may be more likely to occur near busier urban roads, accentuating the bias in the opposite direction (note though that in the Scottish Highlands, many roads cross relatively uninhabited areas). The **35SNP_wildliving_survey_cats** sample represents a contemporary sample of cats that were deliberately targeted for their presence in areas of supposed optimal wildcat habitat (SNH, 2014, Commissioned Report No. 768.), and therefore one presumes might be subject to the same bias as dataset **35SNP_historical_cats**. The **35SNP_historical_cat's** dataset is drawn from a more restricted geographical range, with several cats being collected from the region of the Southern Highlands, thus potentially introducing bias in an unknown direction as there will undoubtedly be both a spatial and temporal component to hybridization.

With these issues in mind, the data are nevertheless strongly indicative that there has been a recent acceleration in hybridization. Hybridization has presumably been occurring to some degree since domestic cats arrived on mainland Britain more than 2000 years ago (Macdonald et al., 2010; Kitchener and Daniels, 2010; O'Connor & Kitchener, 2010), although not in all areas at the same time, but these data appear to indicate that within the last decades it has become considerably more common. The basis for this observation rests on the fact that the historical sample set, which results from targeted sampling of wildcats between 1895 and 1985 (**35SNP_historical_cats**), consists predominantly (54/60) of cats that have an LBQ_35 ≥ 0.75, against the predominantly hybrid contemporary sample sets **35SNP_wildliving_dead_cats** and **35SNP_wildliving_survey_cats** where the majority of cats (87/125 and 19/19, respectively) is defined as genetic hybrids. It is highly unlikely that the distribution of hybrid scores found in the wild in the early to mid‐20th century would have been anywhere close to the continuum seen in road traffic accidents cats since 1990. The distribution of Q_35 scores is significantly (*D* = 0.724, *p*‐value < 0.001) different in **35SNP_historical_cats** to that found in **35SNP_wildliving_survey_cats,** as is the number deemed to be wildcat (Q_35 ≥ 0.75) versus non‐wildcat (Q_35 < 0.75) for management purposes (odds ratio = 0.023, *p* < 0.0001).

The **35SNP_wildliving_survey_cats**, whilst representing a targeted and recent sampling of animals from areas thought to contain wildcats in 2014 (SNH, 2014, Commissioned Report No. 768.), have a distribution of Q_35 scores that is only marginally different to those found amongst the road traffic victims **35SNP_wildliving_dead_cats** (*D* = 0.34737, *p*‐value = 0.03736). No wildcats were sampled in the **35SNP_wildliving_survey_cats**, but this could have arisen by chance due to the small sample size (odds ratio = 0, *p* = 0.0759).

It is worth noting that of the 21 **35SNP_wildliving_dead_cats** that pass the wildcat criteria LBQ_35≥0.75, only two were sampled in the last ten years (since 2008)—two further animals do not have exact dates—the remainder were sampled between 1990 and 2007. More extensive sampling of cats in the wild is currently underway through Scottish Wildcat Action (www.scottishwildcataction.org).

### 4.2 The status of captive wildcats

The higher quality of the captive population could also be taken as evidence to support the temporal trend apparent in the wild samples, as most of the potential founding animals (with known acquisition dates) to the studbook were taken into captivity in 1960s and 1970s although research showing the extent to which all founders are represented today is still in progress. The captive wildcats will have undoubtedly been subject to some level of inbreeding and have certainly been subject to hybridization (9/72 cats are genetic hybrids LBQ35 < 0.75, Table 1), but nevertheless the distribution of scores does not differentiate very significantly from that of the historical cats (*D* = 0.256, *p*‐value = 0.0279). Management measures have since been taken to reduce further hybridization with cats with LBQ_35<0.75 now removed from the breeding population. ddRAD analysis of 100% of the studbook, supported by whole genome sequencing of selected individuals, is in progress to resolve issues with incomplete pedigree data so that future inbreeding can be minimized and a robust long‐term management plan can be developed. It may also be advisable to consider more detailed genetic management through identifying and eliminating introgressed sections of genome (Amador et al., 2014; Amador, Toro, & Fernández, 2012; Hellenthal et al., 2008; Lawson et al., 2012; Malinsky et al., 2016; Price et al., 2009). However, if the level of inbreeding is found to be high, then this will be an equally concerning issue for the Scottish wildcat population in captivity (Crnokrak & Roff, 1999; Frankham, 2010) and genetic rescue (Frankham, 2015) of the population with wildcats from mainland Europe may need to be considered as a long‐term population recovery option. Clearly, any attempt to remove introgressed portions of the genome through captive breeding may also remove important adaptive variation. Inbreeding will also be a threat for populations in the wild as they shrink (Brook, Tonkyn, O'Grady, & Frankham, 2002). These genetic factors will all need to be considered within future restoration programmes for the species.

### 4.3 Measuring hybridization

The interpretation of pelage scores against genetic data is clearly difficult. There is a significant relationship between Q_35 (logit‐transformed) and 7PS pelage (Table 2, Figure 4b); however, the intercept of the relationship varies by dataset (Figure 4), and there is some evidence that the slope may also vary as the interaction of **7PS** with **35SNP_historical_cats** is marginally significant (Est=0.228 ± 0.11, *t* = 2.00, *p* = 0.048). Any discussion of the relationship between 7PS pelage and Q_35 score should be caveated by the statement that the dataset is small (*n* = 104) and therefore we should be wary of over‐interpretation of the available evidence. There is no reason why we should expect a simple relationship between Q_35 and pelage scores, and it is in fact likely that this relationship will be complex (here, fitted quadratic terms to the model were not significant). Phenotypic traits, such as coat colour and the stripe patterning as measured by the 7PS score, are likely to be under the control of a small number of genes (Cieslak, Reissmann, Hofreiter, & Ludwig, 2011; Eizirik et al., 2010), some of which may exert dominant effects on phenotype and which will likely interact to produce pelage traits in a non‐additive manner. As hybridization proceeds to introgression, we expect a halving of average introgressed ancestry per generation of introgression (although there is considerable variance around the mean (Boecklen & Howard, 1997) until a point where hybrids become so common that they mate with each other and complex hybrids are produced. This is clearly the situation within the contemporary wild datasets. Q scores generated by STRUCTURE allow for a relatively simplistic tracking of estimated proportion of ancestry, so that backcrosses cannot be distinguished from more complex hybrids, adding further noise to the relationship since pelage traits will be expected to differ, on average, between backcross generations and other complex hybrid groups. Approaches to classifying the hybrids into generational categories could be applied (NewHybrids; Anderson & Thompson, 2002), but these can fail to generate sufficient resolution between categories of hybrids when both small numbers of markers are used and the systems of hybridization is complex, as is the case here. More detailed genomic data, including the sequencing of candidate genes for pelage traits, may help to untangle this pattern, but at least in the short term, it is hard to see how whole or dense genome data will be able to be used in the making of rapid management decisions in the field or during quarantine, although they may become relevant within future management of the captive breeding programme (see above).

The relationship of 7PS and logit Q_35 displays so much variance that the fine‐scale predictive value of 7PS on Q_35 is low. It seems, however, that using a cut‐off of 17 on the 7PS score broadly sections the least hybridized cats from those that are most hybridized. Of 75 genetically identified wildcats using LBQ_35, 58 would have also been identified by pelage score, with the remaining 17 having a pelage score below 17 (Figure 4c). There were five cats that would pass the pelage cut‐off for wildcat but score below the genetic cut‐off. The 7PS and Q_35 are currently used jointly for ex situ management decisions in a decision matrix where the genetic data carry greater weight (Senn & Ogden, 2015), and it seems that, where logistically feasible, that would be a more appropriate approach to adopt across the board. Currently, only 7PS is used for most in situ management decisions, and by doing so, it seems possible that high genetic‐scoring cats may be missed (Figure 4). Advances in point‐of‐use DNA technology may make this easier over the coming years (Morrison, Watts, Hobbs, & Dawnay, 2018). It should be noted, however, that very few contemporary wild‐living cats have met either the genetic or pelage criteria (Figure 4a). We recommend that the conservation community in Scotland must now define clearly what measures are to be used to define a wildcat living in the wild in Scotland, if future conservation action is to be effective.

## AUTHOR CONTRIBUTIONS

HS conceived and designed the study, analysed the data and wrote the paper. DB and AK analysed the data and wrote the paper. MG and JK performed wet laboratory analysis. BH carried out mapping. RC participated in fieldwork. DWM coordinated survey work. All authors critically reviewed the paper.

## Data Availability

All SNP data available from the Dryad Digital Repository: https://doi.org/10.5061/dryad.1s04tj3.
