# Supplementary figures and images for: Distinguishing the victim from the threat: SNP‐based methods reveal the extent of introgressive hybridization between wildcats and domestic cats in Scotland and inform future in situ and ex situ management options for species restoration
Source: Evol Appl. 2018 Dec 19;12(3):399–414. doi: 10.1111/eva.12720 (PMC6383845; doi:10.1111/eva.12720)

## Slide 1
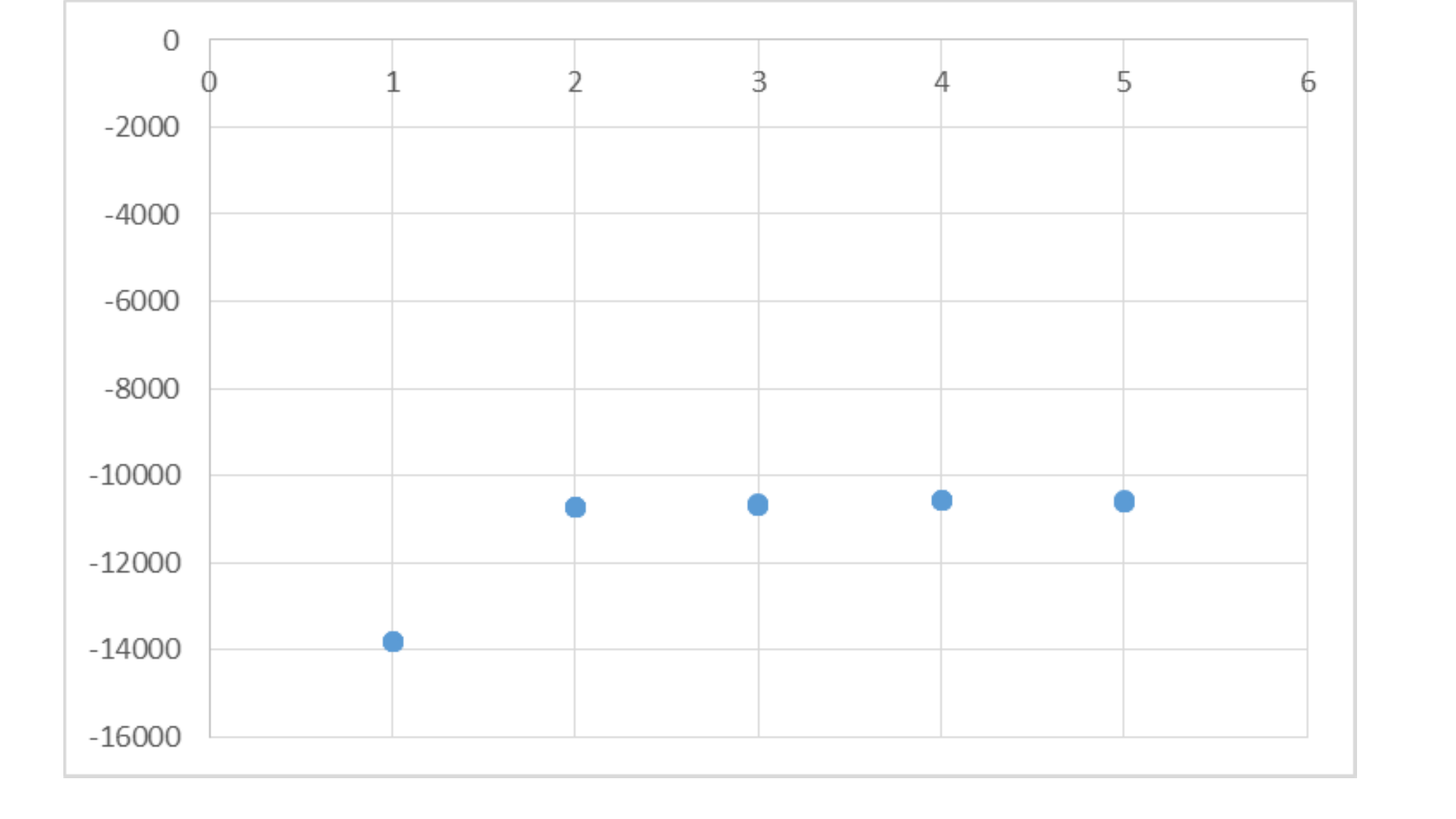

Supplement: Supplementary file 3 [file EVA-12-399-s003.pptx]
